# From Spatial-Temporal Multiscale Modeling to Application: Bridging the Valley of Death in Industrial Biotechnology

**DOI:** 10.3390/bioengineering10060744

**Published:** 2023-06-20

**Authors:** Xueting Wang, Ali Mohsin, Yifei Sun, Chao Li, Yingping Zhuang, Guan Wang

**Affiliations:** 1 State Key Laboratory of Bioreactor Engineering, East China University of Science and Technology (ECUST), Shanghai 200237, China; y20200062@mail.ecust.edu.cn (X.W.); alimohsin@ecust.edu.cn (A.M.); leechao1111@163.com (C.L.); ypzhuang@ecust.edu.cn (Y.Z.); 2 Key Laboratory of Smart Manufacturing in Energy Chemical Process, Ministry of Education, East China University of Science and Technology (ECUST), Shanghai 200237, China; y21220030@mail.ecust.edu.cn

**Keywords:** bioeconomy, hybrid modeling, intelligent biomanufacturing, machine learning, industrial biotechnology, mechanistic model, data-driven model

## Abstract

The Valley of Death confronts industrial biotechnology with a significant challenge to the commercialization of products. Fortunately, with the integration of computation, automation and artificial intelligence (AI) technology, the industrial biotechnology accelerates to cross the Valley of Death. The Fourth Industrial Revolution (Industry 4.0) has spurred advanced development of intelligent biomanufacturing, which has evolved the industrial structures in line with the worldwide trend. To achieve this, intelligent biomanufacturing can be structured into three main parts that comprise digitalization, modeling and intellectualization, with modeling forming a crucial link between the other two components. This paper provides an overview of mechanistic models, data-driven models and their applications in bioprocess development. We provide a detailed elaboration of the hybrid model and its applications in bioprocess engineering, including strain design, process control and optimization, as well as bioreactor scale-up. Finally, the challenges and opportunities of biomanufacturing towards Industry 4.0 are also discussed.

## 1. Introduction

In the wake of agriculture, industry and information economics, bioeconomy is a new economic form that promotes sustainable development globally. Integrating biotechnology and information technology to drive the bioeconomy development is a vital strategy to achieving the target economy and leading a new round of scientific and technological revolution. According to a Forbes 2020 report, bioeconomy accounts for about USD 1 trillion of the U.S. economy (about 5% of GDP (Gross Domestic Product)) [1]. In 2018, the OECD (Organization for Economic Cooperation and Development) released the report—Meeting Policy Challenges for a Sustainable Bioeconomy. This report pointed out the relevant polices for the development of bioeconomy. The United States Congress passed the Bioeconomy Research and Development Act, which established a National Engineering Biology Research and Development Initiative to promote pioneering scientific and technological development. The Chinese government and biological industries are also rapidly developing the bioeconomy. Statistics from the China Commerce Management Institute show that China’s bioeconomy is currently worth CNY 32,905 billion and is expected to reach CNY 50,000 billion by 2025. The bioeconomy places a greater emphasis on coexistence and sustainable development between humans and the environment. Therefore, green and smart manufacturing is transforming the biomanufacturing industry in line with the global goals of green, low-carbon and sustainable development. With the Chinese government’s proposal for “Made in China 2025” and “carbon peaking and carbon neutrality goals” in 2020, biomanufacturing in China is expected to accelerate the transformation towards green manufacturing, which has great developmental potential. Industrial biotechnology produces a wide variety of chemicals, drugs and energy through microbial fermentation, which is an effective mean of solving many of the key problems faced by humanity, including energy and environmental issues [2]. However, industrial biotechnology has long been facing the Valley of Death, where only 1 out of every 5000–10,000 research will successfully turn into commercialized products [3]. To fill this gap, the combination of industrial biotechnology and computation, automation and artificial intelligence has reduced research and development costs., driving a wave of innovation to help speed up crossing of the valley of death. The concept of Industry 4.0, a term introduced by the German government in 2011, is leading to intelligent manufacturing processes, and is fostering profound transformations in the traditional manufacturing landscape. 

**Figure 1:**
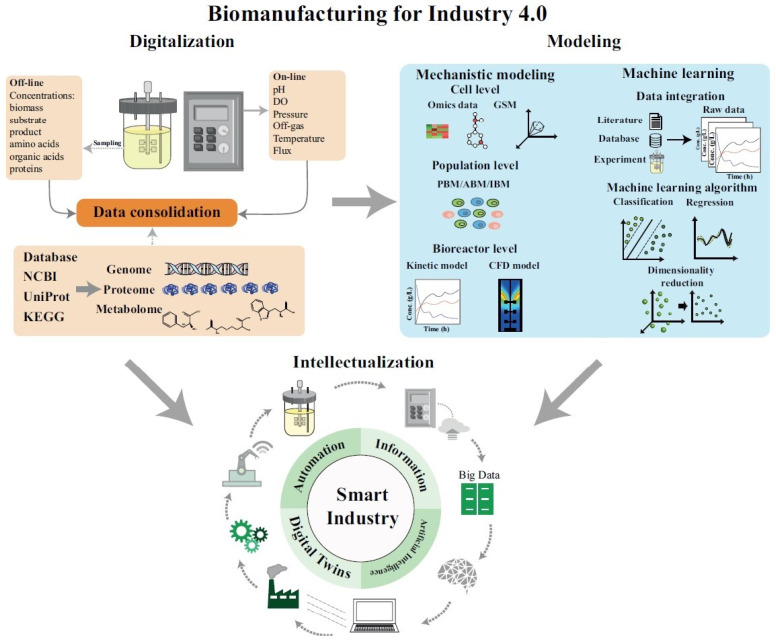
The workflow for biomanufacturing towards Industry 4.0. Digitization: data are obtained through various sources, such as public databases, omics analytics, real-time sensor. Then, these data are integrated to support the construction of models of bioprocess production. Modeling: rational models, which combine artificial intelligence methods with mechanistic models at various spatial scales, are constructed to simulate the bioprocess production based on big data in biological processes. Intellectualization: a digital twin to simulate the industrial biological production process is built based on the big data in biological process and multi-scale hybrid models to improve the production efficiency with automated equipment and finally establish a smart biomanufacturing factory.

For the biomanufacturing industry, there are three main stages to achieve intelligence in manufacturing: digitalization, modeling and intellectualization, as shown in Figure 1. Digitalization is the foundation to achieve intellectualization. Computer, information and communication technologies have been rapidly developed after the third industrial revolution. These advanced devices and digital platforms provide new strategies for big data collection, sharing and analytics during production processes. Data quality is the most important criterion for digitalization. Many methods have been established to improve the quality and amount of data acquisition. For instance, sensor devices enabling real-time monitoring of metabolic transitions of cells during cultivation; rich information resources in public databases such as the NCBI (National Center for Biotechnology Information, https://www.ncbi.nlm.nih.gov/ (accessed on 18 June 2023)) and the KEGG (Kyoto Encyclopedia of Genes and Genomes, https://www.genome.jp/kegg/ (accessed on 18 June 2023)) allow the researcher to design microbial cell factories more precisely and efficiently; continuing advances in multi-omics analytics provide a deep insight into the cellular regulation mechanisms. Modeling is a necessary tool to establish intelligent systems for automating production tasks. Bioprocess models of engineering problems can be established to derive the optimal combination of bioprocess parameters in real-time. Nowadays, many models are available for industrial-scale processes. For example, Monte Carlo tree search (MCTS) is utilized to predict and optimize retrosynthetic routes to guide pathway design in metabolic engineering [4]; computational fluid dynamics (CFD) is a powerful tool to simulate the geometrical and structural properties in the design and optimization of bioreactors [5]; mechanistic models can be established to predict the key process parameters or quality indicators in real-time and thus guide the operations in fermentation processes [6]. Intellectualization is the main feature of automation in production and biomanufacturing, and is also the direction of digital transformation. It aims to achieve automated operation by collecting massive process data in industrial biological processes and analyzing them to monitor and control industrial processes. In this case, researchers can permit timely decision making and effective intervention, and optimize the operations of the equipment within short time periods.

In the last few years, the Internet of Things (IoT) has become increasingly popular in the biomanufacturing industry. The data management and analysis of making informed decisions and optimizing biomanufacturing processes is the main bottleneck. In the digitization process, some data is redundant and disordered. Omics data can represent cellular changes in biological processes at various levels, such as transcriptional regulation, protein expression, and metabolic regulation. However, there are few studies that have combined multi-omics data with production operations to analyze metabolic changes comprehensively across multiple scales. Furthermore, industrial bioprocess data are sparse and high-dimensional, so it is important to use dimensionality reduction to tackle problems on the premise of guaranteeing the data quality. In the modeling process, different models have been constructed to cater to the practical needs of different engineering problems. Genome-scale metabolic models (GEMs) are well proven tools for the in silico analysis of microbial physiology for designing and optimizing metabolic flux distributions in genetic engineering by flux balance analysis (FBA). Unfortunately, due to a lack of capacity to capture the full annotation of omics datasets, the applications of GEMs are limited in guiding cell engineering [7]. Kinetic models, mathematical descriptions of dynamic metabolic systems, aim to estimate the key process parameters in industrial production [8]. However, the construction of kinetic models requires a large amount of process data and detailed biochemical process mechanism of the engineering object, which is the reason that the workload of the construction of mechanistic model is high. Additionally, the structural and quantitative uncertainty surrounding kinetic representations is also a key challenge due to incomplete knowledge of regulatory interactions and the high dimensionality of rate laws [9]. By contrast, data-driven models have outstanding ability in dealing with large and complex data in industrial production through machine learning methods. These kinds of models can be used to simulate the link between cellular metabolism and bioreactor operations without a comprehensive knowledge of related mechanisms [10]. However, data-driven models rely on the quality of historical data and lack the interpretation of biochemical mechanisms, so large deviations will be formed when the raw data are noisy. 

Hybrid models are models that combine mechanistic models and machine learning algorithms (data-driven models) with bioprocess information at multi-spatial and temporal scales. Such hybrid models can compensate for the lack of biological mechanisms in data-driven models and the large workload of mechanistic models. Interestingly, machine learning is a kind of artificial intelligence with a wide range of applications, such as natural language understanding [11], image recognition [12], autonomous driving [13] and medical diagnosis [14], as well as the bioprocess engineering industry. For example, deep neural networks accelerate the design-build-test-learning (DBTL) cycle in metabolic engineering by predicting and optimizing targeted pathways [15]. This method makes progress by developing new cell factories that meet the economic requirements for industrial scale production. Furthermore, the computational fluid dynamics (CFD) models coupled with convolutional neural networks (CNN) can help optimize the configuration of bioreactor operations and reduce computational costs from months to days [16]. 

This review summarizes the recent developments of mechanistic and data-driven models, and their applications in bioprocess engineering. We mainly focus on the applications of hybrid models in bioprocess development, including the design and optimization of engineered strains, monitoring and control of biological production processes and simulation and design of bioreactors. In addition, we will also discuss the current challenges and future perspectives for the development in biomanufacturing.

## 2. Development of Modeling

### 2.1. Mechanistic Models

**Figure 2:**
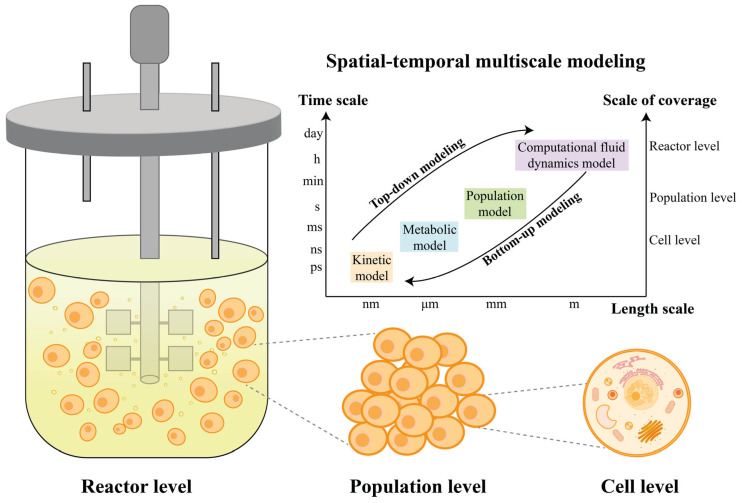
Spatial–temporal multiscale modeling. The three spatial scales are depicted as reactor level, population level, and cell level. The modeling at reactor level mainly focuses on the overall dynamic behavior including mass transfer, flow characterizations, bubble and particle behavior by CFD models. The modeling at population level considers growth and competition among individuals and the interactions between individuals and environment. At the cell level, the intracellular biological process, such as metabolic networks, signal transduction pathways and gene regulation mechanisms are predicted by metabolic models.

Mechanistic models are mathematical models established based on the mechanisms of the production host and its production process. Mechanistic models describe the dynamic process using mass and energy balances [8]. Mechanistic models play an important role in comprehensively exploring the reasons for changes in cell growth and production across multiple scales, from genetic to cellular to bioreactor levels, as shown in Figure 2. Broadly, the mechanistic models can be divided into two main categories: unstructured cell models and structured cell models. An unstructured cell model is regarded as a “black box” as it describes cellular growth and production using a single state without considering intracellular metabolic events. A structured cell model considers the reactions between intracellular states and environments. Mechanistic models can also be classified as unsegregated and segregated models. An unsegregated model describes the behavior of cells as an average behavior in bulk while a segregated model considers heterogeneity among the cell population. In this section, we mainly focus on kinetic models, GEMs and CFD models. 

#### 2.1.1. Unstructured Unsegregated Models

Unstructured kinetic models can describe changes in main state parameters such as biomass, substrates and products to predict optimum process conditions for production. In 1913, Michaelis and Menten proposed a hyperbolic relationship between the enzyme-catalyzed reaction rates and the concentration of the substrate, which was regarded as the Michaelis–Menten equation [17]. Michaelis investigated the effect on enzyme-catalyzed reaction rates of temperature and pH [18]. In 1942, Monod, the founder of cellular growth kinetics, proposed a hyperbolic relationship between the concentration of substrate and cell growth kinetics (i.e., the Monod model, see Equation (1)) [18]. The Monod kinetic model describes the growth kinetics of cells through thousands of enzymes, which is the most widely used unstructured kinetic model [19]. For example, Pau et al. described the uptake rates of the substrates, glucose and xylose, and the inhibitors using a Monod-type kinetic model in the lignocellulosic fermentation [20]. However, the Monod model cannot assess the relationship between specific growth rate and substrate utilization with an excess of substrate. At a high substrate concentration, the specific growth rate may be hindered by the presence of toxic substrate. Another limitation of the Monod model is that it does not take into account the lag and death phase during the growth phase. In the past few decades, many kinetic models have been developed to address the limitations of the Monod model. For instance, the Haldane model (see Equation (2)) introduced an inhibition constant (*K_I_*) to deal with specific growth rate inhibition at low and high substrate concentration; the Aiba-Edward model (see Equation (3)) is capable of describing the lag and death phase [21]. More information about the development of unstructured kinetic models can be found in the following references [9,21,22].

(1) $$\begin{array}{c} \mu =\mu_{max}\frac{S}{K_{S}+S} \end{array}$$

(2) $$\begin{array}{c} \mu =\mu_{max}\frac{S}{K_{S}+S+\frac{S^{2}}{K_{I}}} \end{array}$$

(3) $$\begin{array}{c} \mu =\mu_{max}\frac{S}{K_{S}+S}exp\left(-\frac{S}{K_{I}}\right) \end{array}$$

#### 2.1.2. Structured Unsegregated Models

The structural kinetic models investigate the dynamic changes in the specific metabolic pathways (such as the glycolysis pathway) in response to the culture conditions. For instance, Cronwright et al. constructed a kinetic model of glycerol synthesis via glycerol 3-phosphate, presenting details on the kinetics of the enzyme-catalyzed reactions by metabolic control analysis [23]. This model might shed some light on the inherent capacities of the pathway and guide controlled glycerol synthesis by *S. cerevisiae* in industrial production [23]. In penicillin production, Tang et al. developed a 9-pool metabolic model by lumping the most important intracellular metabolites into five pools and four intracellular enzyme pools [24]. This model can describe the dynamics of cell growth, penicillin productivity and intracellular metabolite pools under a periodic glucose feast-famine cycle experiment at time scales from minutes to days [24]. The construction of kinetic models requires determinations of the basic kinetic parameters from a set of ordinary differential equations (ODE) by existing software tools such as Systems Biology toolbox [25], a MATLAB software toolbox. Relevant kinetic, thermodynamic, and stoichiometric information also need to be integrated. To simulate the structure and parameters of the kinetic model more accurately, researchers rely on public databases like BRENDA [26] and SABIO-RK [27], combined with multi-omics data and a wealth of phenotype data [28,29,30,31,32]. As a leading example, Cotton et al. determined the reaction direction and crucial kinetic parameters in the model with proteomic, metabolomic and thermodynamic data, leading to a more accurate estimation of the growth and metabolic fluxes of the central carbon metabolism in *Escherichia coli* [33].

Motivated by the development of these models, methods to construct large-scale kinetic models of metabolism have started to emerge. Genome-scale metabolic models (GEMs) provide valuable insights into the functioning of metabolic networks and mechanisms associated with cell growth and product formation, aiding in the construction of large-scale kinetic metabolic models.

The GEMs transform the relationship among genes, enzymes and metabolites in the process of cell growth and metabolism into a set of mathematical equations based on a stoichiometric matrix to simulate the metabolic fluxes [34]. Nowadays, GEMs have become well-proven tools for in silico analysis of cellular metabolism. GEMs have been widely used in various industries such as pharmacy, food and chemistry to explore metabolic phenotypes, analyze metabolic mechanisms and guide metabolic design [35]. Since the first GEM of *Haemophilus influenzae* Rd was reported in 1999, the reconstructions of GEMs for 6239 organisms (5897 bacteria, 127 archaea, and 215 eukaryotes) have been built, including various industrial model organisms such as *Escherichia coli*, *Saccharomyces cerevisiae* and CHO cells [36,37,38,39,40].

Flux balance analysis (FBA) has been widely used to explore cell growth in steady state by GEMs, while dynamic flux balance analysis (DFBA) has been further developed to simulate cellular phenotypes in dynamic state [41,42]. The development of automated tools such as ModelSEED [43], MetaMerge [44] and MEMOTE [45]; integrated tools such as COBRA TOOLBOX [46] and Raven [47]; and rich resources available in public databases such as BRENDA [26], KEGG [48] and MetaCyc [49] has greatly aided the constructing of multi-scale constraint-based GEM. For example, O’Brien et al. constructed a ME-Model for *Escherichia coli* (ME-MG1655), computing ~80% of the functional proteome to predict multi-scale phenotypes and mimic the transcription and translation capabilities of cells in a given steady-state environment [50]. The cellular metabolism depends not only on the gene-protein-reaction (GPR) relationship, but also is influenced by the external environment. Thus, the constrained metabolic models (CBMs) based on the typical GEMs improve the accuracy of predictions of the cellular phenotypes effectively. Metabolic reactions inside the cell conform to the laws of thermodynamics, so the direction of metabolic reactions is determined according to the change in the Gibbs free energy. For example, Henry et al. improved the accuracy of the estimation of kinetic parameters in the iJR904 genome-scale metabolic model of *E. coli* based on the group contribution method that determined the thermodynamic feasibility of the reactions [51]. Moreover, the kinetics of the functional enzyme is also important for cellular metabolism. Many tools have been built to establish enzyme-constrained GEMs that integrated proteomic data to explore the effect of the enzyme usage on phenotypes in the metabolic process. For example, a novel metabolic network-based approach, Metabolic Modeling with Enzyme kinetics (MOMENT), predicts metabolic flux rate and growth rate through enzyme turnover rates (k_cat_) and enzyme molecular weights [52]. GECKO (a method that enhances a GEM with Enzymatic Constraints using Kinetic and Omics data) integrates enzyme kinetics (k_cat_) and quantitative proteomics (protein abundances) to constrain a GEM, correctly representing capacity constraints on fluxes [53]. This approach provides insights into the enzyme usage of each metabolic reaction, and has confirmed its good performance in *S. cerevisiae*, significantly resulting in a decrease in flux variability. Subsequently, the GECKO toolbox was updated in its 2.0 and 3.0 version with the improvement of its parameterization procedure to ensure high coverage of kinetic constraints, expanding its use for building enzyme-constrained models (ecModels) for more organisms [53,54]. Nevertheless, enzyme-constrained models cannot simulate cellular growth under environmental perturbations, while imposing kinetic constraints to GEMs (kinetic constraint GEMs) captures the change of kinetic parameters of enzymes under environmental perturbations, delving deeper into the variations of metabolic phenotypes [55]. Towards this aim, toolkits have been developed for constructing such kinetic constrained GEMs, such as structural kinetic modeling (SKM), the mass action stoichiometric simulation (MASS), optimization and risk analysis of complex living entities (ORACLE), ensemble modeling (EM) and approximate Bayesian computation-general reaction assembly and sampling platform (ABC-GRASP) [34]. An in silico approach to reduction of characterization in uncertainty in the kinetic models of genome-scale metabolic network (iSCHRUNK) based on the ORACLE framework was further developed to identify the key enzymes in the metabolic network and quantify the kinetic parameters to increase the accuracy of the model [56]. In addition, owing to the complex metabolic mechanism, a comprehensive analysis of multi-omics data from different scales allows in-depth understanding of the metabolic mechanisms that regulate cellular growth and production. For example, Huang et al. integrated time-series transcriptomic data into GEMs of CHO cells, comparing various growth stages of different cell lines and using datasets from one cell line to leverage cell growth condition in other cell lines [57].

Although the GEMs have been widely used to simulate complex metabolic events, the dynamic environment experienced by the cells in the large-scale bioreactor also induces metabolic heterogeneity. Hence, taking into account the cellular heterogeneity caused by the changing environment in the bioreactor, computational fluid dynamics (CFD) models are a vital tool to analyze the effects of cellular metabolic characteristics and the external environment [58].

#### 2.1.3. Segregated Models

CFD models have played a significant role in the design, optimization and process scale-up, providing detailed flow field information in the bioreactors. The main methods for CFD modeling of multiphase flow are divided into Euler-Euler (EE) modeling and Euler-Lagrange (EL) modeling. Due to its low computational cost, the EE method has been widely applied to study the effect of operating conditions and geometry on the flow field structure and substance concentration distribution in industrial production [59]. The EL approach tracks the movement trajectory of each cell particle in the flow field, so it can reproduce how the bioreactor environmental heterogeneity affects the cellular metabolism [60]. For example, the gas-liquid mass transfer in the stirred bioreactors was simulated to analyze the effect of the size and shape of the gas bubbles and oxygen mass transfer on the utilization of oxygen in aerobic biological fermentation process [61]. Sarkar et al. studied the effects of stirring paddle speed and ventilation rate on bubble coalescence and rupture [62]. They finally found the optimal stirring and ventilation strategy of the bioreactor in the process of monoclonal antibody production in animal cell culture [62].

The population balance model (PBM) is a segregated model that describes the growth and distribution of cells, as well as the differentiation of cells caused by environmental changes. Morchain et al. used the two-phase Euler-Euler method and the PBM model based on the cell-specific growth rate to simulate the gas-liquid mixing and cell distribution in the laboratory scale and industrial scale production [63]. They discovered that the main bottleneck of scale-up was the spatial heterogeneity of the specific substrate consumption rate and specific growth rate of cell subsets [63]. Similarly, Pigou et al. combined PBM and metabolic models to explore the relationship between the environmental changes and the heterogeneity of *E. coli* cells [64]. The researchers found that due to poor mixing in the industrial bioreactors, a glucose concentration gradient formed and induced the differences in acetate production and consumption levels in different regions of the reactor [64]. They successfully explored the reasons of yield declines and by-product formation in large-scale production [64]. The agent-based model (ABM) considers cells as individuals which move constantly in a heterogeneous environment, capturing the interaction between individual cells and the environment. The ABM model aids in identifying the structure of cell populations, understanding the metabolic heterogeneity. The ABM model can be used to simulate the spatiotemporal dynamic changes of cell populations under disturbance at different times and spatial scales [65]. Lapin et al., firstly, researched the dynamic response of *S. cerevisiae* cells under glucose disturbance with the combination of the cell motion trajectory and the flow field [60]. They finally successfully simulated the dynamic interaction between yeast cells and the spatial heterogeneous environment in a laboratory-scale reactor by Euler-Lagrange method [60]. Then, the researchers studied the effect of different glucose concentrations on the metabolism of *E. coli* in a 900 L bioreactor using this method [66].

Considering the interaction between cells and the flow field environment, the kinetic models with cell dynamics information and the metabolic network model should be combined in order to simulate the actual fermentation process. Du et al. combined CFD and kinetic models to simulate the biomass growth, lipid accumulation and the flow field environment during the production of docosahexaenoic acid (DHA) by fission yeast [67]. They explored the optimal process conditions and validated the DHA production performance in a 35 m^3^-scale bioreactor [67]. The results served to propose an efficient industrial bioprocess scale-up strategy [67]. Liu et al. discovered that by combining CFD with cell death kinetics the maximum shear stress and shear frequency (SSF) parameter could effectively reflect the relationship between shear environment and cell death rate during the scale-up of *Carthamus tinctorius* L. cells in a 15 L STR bioreactor [68]. Haringa et al. coupled the 9-pool metabolic model of *Penicillium chrysogenum* with CFD to track the movement trajectory of cells in a large-scale production and evaluate the impact of flow field changes on yield [69]. Moreover, Haringa et al. considered the compartment model and tracked the intracellular response to extracellular changes during production using a stochastic parcel tracking approach [70]. This greatly reduced the calculation time of the model and effectively improved the computing capacity [70]. 

Currently, the main bottleneck of the hybrid models is the large computational cost required for the simulation of the large-scale microbial metabolism and the long-term production processes. Forms like lattice Boltzmann (LB) and dynamic large-eddy simulation (LES) have been developed to reduce the computation times [71]. Haringa et al. validated the performance of lattice Boltzmann large-eddy simulations (LB-LES) in the bioreactor when resolving substrate gradients in the penicillin production, which provided guidance for rational design and scale-down of the large-scale bioreactors [72]. Witz et al. simulated the flow fields and bubble movements by the lattice Boltzmann method (LBM) and Lagrange approach, where the distribution of bubbles in 40 m^3^ bioreactors provided importance clues for designing bioreactors at an industrial scale [73]. Nevertheless, for complex production processes at an industrial scale, the computational time may still reach one week or even longer. In such scenarios, data-driven models that use machine learning methods and are efficient in data processing can enhance the accuracy of CFD models in a more efficient way.

### 2.2. Data-Driven Modeling

**Figure 3:**
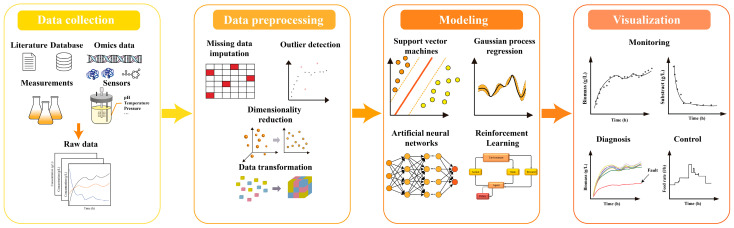
Workflow of the construction of data-driven models. It consists of 4 steps including data collection, data preprocessing, modeling and visualization. Data collection: big data are collected in various ways, such as omics data from databases, the operation variables from online sensors and then integrated to the raw dataset. Data preprocessing: the raw dataset is preprocessed into a standard format for model construction. Modeling: machine learning algorithms are used to construct the data-driven models through the big data collected in bioprocess to predict the key variables. Visualization: the model outputs are visualized to improve interpretability.

Data-driven models are based on big data and collect information from multiple sources, including omics data, state variables of fermentation processes sampled by online sensors and other resources from public databases. Data-driven models approximate the input–output relationship without considering the underlying mechanisms of biological processes, treated as black boxes. Data-driven models make full use of big data gathered in the historical fermentation process to guide the operations in the industrial scale production, as shown in Figure 3. Data-driven models have become a research hotspot compared to mechanistic models due to their simpler structure and fewer parameters. A quick search of the literature was undertaken using the search words “Machine learning” and “fermentation” on Web of Science. A total of 65 research articles primarily published from 2000 to 2022 have been selected. By analyzing these publications, the most popular machine learning methods are artificial neural networks (124), support vector machines (98), multivariate statistical analysis (59) and random forest (43). In this section, we focus on the introduction of these most widely used machine learning methods, support vector machines (SVM), artificial neural networks (ANN), Gaussian process (GP) and reinforcement learning (RL), and their respective characteristics as well as application scenarios are discussed (Table 1). 

#### 2.2.1. Support Vector Machine (SVM)

Support Vector Machines (SVMs) are a well-established technique, based on statistical learning that analyzes complex bioprocess data with high nonlinearity and time-varying in biological fermentation, they have been widely used to construct soft-sensor models in the biological development process [74]. For instance, Li et al. used SVM to predict the penicillin titer in real-time in the industrial production [75]. Du et al. constructed a multi-kernel SVM to predict the average molecular weight in the polyacrylonitrile productive process, better than the performance of single-kernel SVM [76]. Zhang et al. established a soft-sensor model of microbial lipids from cellulosic ethanol wastewater by *Rhodotorula glutinis* to optimize the operation parameters with genetic algorithm (GA), and finally improved the maximum biomass and lipid production to 11.87 g/L and 2.18 g/L, respectively [77].

Furthermore, many improved SVMs combined with other advanced algorithms have been developed to improve the performance and widen the range of applications. For instance, Jin et al. successfully increased the titer of penicillin by 22.88% using a combination of real-time coding genetic algorithm (RGA) and SVMs [78,79]. Urtubia et al. combined Particle Swarm Optimization (PSO) and SVM to identify and diagnose the abnormal markers in wine fermentation, and greatly improve the accuracy to classify the abnormal batches in the early 72 h [80]. However, due to the weak capability to deal with large datasets, the application of SVMs has been limited with big datasets.

#### 2.2.2. Artificial Neural Network (ANN)

Artificial neural networks (ANNs) are nonlinear, adaptive information processing systems consisting of a large number of interconnected processing units. ANN is an effective tool to identify the non-linear relationship between fermentation parameters (inputs) and biological parameters (outputs), which are highly non-linear changeable in the fermentation [81]. Nowadays, ANN has been widely used in predicting important state variables and optimizing processes, etc. [82,83,84]. ANN consists of an input layer, a hidden layer and an output layer. The parameters of the neural network are iteratively updated by the neuron nodes in the hidden layer and their weights to predict a specific quantity (output value), as:

(4) $$\begin{array}{c} y=f\left(\sum_{i=1}^{n}w_{i}x_{i}+b\right) \end{array}$$

where *w* represents weights; *x* is arbitrary inputs; *y* is outputs; *b* is the bias value and *f* is the activation function. The activation function plays an important role in ANN, which is the learning method of neural networks, like the sigmoid function (Equation (5)) and the ReLU function (Equation (6)).

(5) $$\begin{array}{c} f\left(x\right)=\frac{1}{1+e^{-x}} \end{array}$$

(6) $$\begin{array}{c} f\left(x\right)=max\left(0,x\right) \end{array}$$

Weight values and thresholds in neural networks are updated using forward and backward propagation techniques. Backward propagation neural networks with sigmoid functions have been widely used for modeling and optimizing biological processes [85]. For example, Peng et al. predicted the antibiotic effect of bacteriocin 1701, and further optimized the fermentation parameters using a time-dependent ANNs strategy with genetic algorithm (GA) [81]. This approach eventually increased the production yield by 26% [81]. Ding et al. developed an adaptive feeding control system using ANN to recognize glucose depletion faults in real-time during glutamate fermentation to realize feeding glucose automatically [86].

Neural networks can be classified into different types based on their structures. The following networks have been widely used in various fields: convolutional neural networks (CNN), deep neural networks (DNN), recurrent neural networks (RNN), long short-term memory networks (LSTM) and generative adversarial networks (GAN). Among them, RNN and CNN are the two most widely used types. For example, the advanced image recognition processing capabilities of convolutional neural networks are applied in various fields of biological process development. AlphaFold, which is based on CNN, are capable of predicting protein structures [16]; the U-Net CNN is used to automate the counting of bacterial colony forming units (CFUs) and distinguish virulent colonies from avirulent colonies in vaccine development [87]. Meanwhile the CNN has also been applied to predict microalgae production and optimize process operating conditions [88]. Recurrent neural network (RNN) is an artificial neural network for series data, which can transfer information between neurons and express the correlation between data while taking the time dimension into account. RNNs have been widely used in the time-series prediction of key state variables and diagnosing faults in fermentation, particularly in industrial-scale production [89,90]. Beiroti et al. accurately predicted the biomass of recombinant *Pichia pastoris* Mut^+^, and optimized the process conditions in the methanol induction phase of the fed-batch fermentation [91]. This guidance led to the large-scale production of intracellular hepatitis B surface antigen (HBsAg) [91]. However, RNN meets the problem of gradient disappearance and gradient explosion in the process of modeling with long-term datasets. Therefore, a long-short memory network (LSTM) is developed to construct long-term time-series dependent models. Yuan et al. constructed a soft-sensor model to predict the product titer of penicillin by a supervised LSTM network (SLSTM), which significantly improved the accuracy compared to the model based on RNN [92]. The performance of neural network models depends on a large number of datasets. Thus, the augmentation of data sets can significantly relieve the pressure from the data acquisition of the production with a small sample size [93]. Interestingly, GAN is used as an alternative strategy. For instance, Wang et al. proposed DA-GAN (dual adversarial learning-based virtual sample generation method) to generate the same distributions of real data from industrial processes [94]. This method was also applied in industrial cases, solving the challenge of a shortage of data [94].

#### 2.2.3. Gaussian Process (GP)

Gaussian processes (GPs) are a probabilistic machine learning method based on statistics. The prediction result of GP is given by Gaussian distribution. The mean value of the distribution can be regarded as the prediction value while its variance is regarded as the uncertainty range of the result. The quantification of the uncertainty makes GP a powerful tool within biological process control. For example, Mei et al. established a soft-sensor model for erythromycin fermentation at an industrial scale with principal component analysis (PCA) which focused on selecting important features to simplify the model structure [95]. This method has excellent prediction performance for biomass concentration in the exponential growth period of erythromycin fermentation [95]. Zhang et al. optimized the process in phycocyanin production by cyanobacteria in a semi-batch bioreactor through nonlinear model predictive control (NMPC) and successfully constructed the optimal nitrate feed strategy in the actual plant production process [96]. GPs can be regarded as the generalization of multivariate Gaussian distribution, which is determined by mean function $\mu \left(x\right)$ and covariance function $k\left(x,x^{*}\right)$, as:

(7) $$\begin{array}{c} f\left(x\right)\sim GP\left(\mu \left(x\right),k\left(x,x^{*}\right)\right) \end{array}$$

Gaussian processes directly output the probability distribution and the confidence interval of the prediction, which make the optimization of process control more stable and realistic in large-scale fermentation production. Nevertheless, as a non-parametric model, the covariance matrix inversion of all data points is required for each operation, which greatly increases the calculation cost. Hence, it is not suitable to deal with large-scale data sets, or apply in the industrial production process of non-Gaussian process.

#### 2.2.4. Reinforcement Learning (RL)

Reinforcement learning (RL) is different from unsupervised learning and supervised learning, as a machine learning approach seeking optimal control strategies. Although game-based control has frequently employed reinforcement learning, its applicability in biological process engineering has been limited until now [97]. Since reinforcement learning can elaborate process stochasticity and nonlinear dynamics, it has great potential in optimal control, production scheduling, and so on. It has been applied in process controlling in fed-batch fermentation and de novo design of drugs and proteins [98,99]. For example, Li et al. constructed a multi-objective reinforcement learning method to control the feeding operation in the lysine fed-batch fermentation [100]; Pandian et al. proposed a partially supervised reinforcement learning (PSRL) control strategy to regulate the substrate concentration in Baker yeast fermentation and realize the liquid-level control in MIMO (Multiple-Input Multiple-Output) quadruple tanks with Q-learning functions [101].

The reinforcement learning framework treats the control problems as an optimal sequential decision problem referred to as Markov Decision Process (MDP), including agent, environment, state, action and reward. After an agent performs an action, the environment state is updated, and the new state sends a reward signal to the agent. Subsequently, the agent performs a new action based on the new state and the reward signal. Thus, reinforcement learning does not require detailed mechanistic knowledge to learn the strategy, but recalibrates the strategy with data changes to obtain the optimal strategy. The RL is divided into two categories, model-based reinforcement learning and model-free reinforcement learning. Model-based reinforcement learning is based on model-based environments where policies can be developed in advance, such as model predictive control (MPC) [102]. MPC solves control problems using optimization methods, such as variational methods and dynamic programming but the associated computational cost is very high. For example, Lee et al. constructed a two-stage fed-batch control framework by a model-based reinforcement learning algorithm (differential dynamic programming, DDP) with model predictive control (MPC) to optimize the feeding operations in penicillin production [103]. On the one hand, model-based reinforcement learning requires continuous updating of datasets, leading to large errors if the model data do not match the actual situation. When the simulation of complex biochemical metabolic processes causes high computational costs, large errors occur with low-precision models. Model-free reinforcement learning, on the other hand, obtains optimal strategies through real-time interaction between the agent and the environment without requiring precision. For example, Lee et al. integrated MPC with the double-deep Q-network algorithm to obtain the optimal substrate feeding strategy in industrial-scale penicillin production, effectively reducing the operating cost of semi-intermittent bioreactors [104]. Benton et al. optimized the feeding process for cyanobacterial-phycocyanin (C-PC) production by the Asynchronous Advantage Actor-Critic (A3C) algorithm with asynchronous learning control and finally increased the product yield by 52.1% [105]. To confirm the optimal policy, model-free reinforcement learning requires a large number of interactions with the environment to achieve the desired learning effect. A large number of training samples and training time are the main bottleneck in application in the industrial production.

**Table 1:** Pros and cons of different algorithms for the construction of data-driven modeling [106].

Method	Advantages	Disadvantages
Support vector machine (SVM)	Suitable for high-dimensional datasets; Suitable for solving non-linear problems; Various kernel functions for different problems.	Not suitable for large datasets; High requirements on data; Preprocessing and selections of hyperparameters.
Artificial neural network (ANN)	Suitable for solving non-linear problems; Robustness to noise; Suitable for large datasets.	High requirements on the integrity of datasets; Hyperparameter optimization at a high computational cost; Poor generalization capability.
Gaussian process (GP)	Suitable for solving non-linear problems; Capacity of predictive values and their uncertainty; Various kernel functions for different problems.	Not suitable for large datasets; High computational costs.
Reinforcement learning (RL)	Suitable for decision problems in time-series models; Suitable for optimization problems; Good generalization capability.	High requirements on data quantity and quality; Difficulty to design the reward function.

### 2.3. Multi-Scale Hybrid Modeling

The entire bioprocess development, from strain design to industrial production, spans multiple spatial and time scales. Importantly, optimizing each step of bioprocess development requires a comprehensive understanding of cell growth, production and the key factors involved. This can be achieved through a hybrid model that links dynamic information across scales.

The GEMs describe the metabolic mechanisms of cells at steady state, while the actual production process is a constantly changing process. To explore metabolic phenotypic changes from genome-scale to cell scale, a hybrid model that integrates GEMs with the kinetic model is required. CFD models can predict the flow field changes in bioreactors and analyze the effects of environmental perturbations on cell metabolism, even at the industrial production scale. The hybrid models that combine CFD models with kinetic models to reproduce the cellular production process from multi-spatial scales provide an opportunity to further investigate the mechanisms of metabolic phenotypic changes during cell growth and production. For instance, Haringa et al. coupled a CFD model with a 9-pool metabolic model to assess the effect of substrate heterogeneity on industrial-scale production of penicillin, providing guidance for rationally designing scale-down models [69].

Mechanistic and data-driven modeling are the main two crucial techniques for bioprocess model construction. Hybrid models that combine data-driven and mechanism models greatly reduce the cost of model construction and improve efficiency. For example, a hybrid model that integrated the Contois model and Gaussian process (GP) had higher accuracy in the prediction of the production of astaxanthin by *Xanthophyllomyces dendrorhous* than the kinetic model [107]. The uncertainty of the hybrid model was decreased with the percentage standard deviation from 15.5% to 8.6% for biomass and from 13.2% to 9.17% for astaxanthin [107]. The following section will specifically focus on the role of hybrid models in the fields of metabolic engineering and bioprocess engineering.

## 3. Applications of Hybrid Models in Bioprocess Development

### 3.1. Metabolic Engineering

Despite the advancements in the system and synthetic biology, developing new cell factories by traditional metabolic engineering remains challenging. It typically requires several months or even years to meet the economic requirements for industrial-scale production [15]. Recently, researchers have utilized advanced machine learning methods and omics technology to construct models that simulate complex cellular metabolism. These methods and technologies aid in improving the accuracy of product synthesis pathway design and optimization of metabolic flux, while significantly reducing the cost of research and development.

#### 3.1.1. Metabolic Model Reconstruction for Better Performance

The GEMs are an important tool to investigate cell growth and production, so continuously upgrading the models by supplying the missing information of the metabolic network to improve the accuracy of the GEMs is essential. With the development of omics technology, rich information on genomics, transcriptomics, proteomics and metabolomics has provided a detailed supplement in the reconstruction of metabolic pathways. For example, Sánchez et al. applied GECKO to a *Saccharomyces cerevisiae* GEM (ecYeast7) by integrating kinetic and omics data to constrain the proteome resource allocation with an enhanced performance on phenotype prediction [53]. Nielson et al. developed a deep learning method (DLk_cat_) based on graph neural network (GNN) and convolutional neural network (CNN) [108]. DLk_cat_ integrated substrate structure information and protein sequence information to achieve high-throughput prediction of k_cat_ of cell metabolic enzymes [108]. This method was applied to reconstruct 343 ecGEM models of yeasts [108]. Culley et al. proposed a multimodal learning framework based on transcriptomics and fluxomics to predict the growth phenotype of *S. cerevisiae* cells with the integration of large-scale gene expression profiles and mechanistic metabolic model constrained based on transcriptome data [109]. A multi-view neural network method was used to compare the performance of the multi-omics constrained GEMs [109]. This method increased the prediction accuracy and provided tools for understanding the relationship between the biological mechanisms of metabolic changes and the phenotypes [109].

Identifying EC numbers to determine enzyme function is essential for identifying key enzymes in metabolic pathways to design and optimize target metabolic pathways. Ryu et al. developed DeepEC, a tool based on convolutional neural network (CNN) which takes protein sequences as input and EC numbers as output, to predict EC numbers with high precision and throughput [110]. Protein engineering methods are used to design new enzymes to meet metabolic requirements when key enzymes are missing in the target metabolic pathway. Directed evolution is a common approach to protein engineering, involving high-throughput screening of enzymes by iterative point mutation. However, this approach is associated with an enormous workload (for example, for a protein of 300 amino acids, there are 5700 single-point mutations and 32,381,700 double-point mutations) [111]. Deep learning methods such as variational autoencoder (VAE) and generative adversarial networks (GAN) can effectively improve the efficiency of predicting protein function and generating protein sequences with new functions to achieve rational protein design [111].

#### 3.1.2. Metabolic Model-BASED Guidance for Strain Design

After designing a reasonable metabolic pathway, it is necessary to optimize metabolic flux allocation, identify key metabolic fluxes and maximize product titer, rate and yield (TRY) [112]. Metabolic flux analysis (MFA) allows kinetic models to simulate large-scale dynamic metabolic pathway fluxes so that the research cost can be greatly reduced. This method has been validated by simulating glycolytic reaction fluxes in *Escherichia coli* and human red blood cells [113]. Starepravo et al. proposed a hybrid model that integrated the kinetic model and dynamic MFA to simulate the flux change in the batch fermentation process with the combination of a single-level mixed-integer quadratic program (MIQP) [114]. This model can identify the shortest metabolic pathway from substrate to product, which has been applied in the biosynthetic pathway for astaxanthin production in *Saccharomyces cerevisiae*, reducing the original metabolic network by 70% [114]. Carinhas et al. updated a stoichiometric model to identify the key metabolic pathways involved in baculovirus production in insect cells by partial least squares (PLS) and MFA [115]. They finally targeted the TCA cycle and mitochondrial respiratory pathways as the key pathway to virus replication, guiding for the feeding operation optimization [115]. Precisely optimizing multi-gene metabolic pathways is a major challenge in metabolic engineering. HamediRad et al. constructed a fully automated robotic platform, named BioAutomata, using an integrated robotic system coupled with machine learning algorithms in order to fully automate the DBTL process for biosystems design [116].

### 3.2. Bioprocess Engineering

Hybrid modeling is an effective tool for the prediction of the key state variables in the bioprocess to explore the relationship between the operating parameters of the bioreactor and cell metabolism. Additionally, with advanced biosensors, the bioprocess can be monitored in real-time, which is beneficial to the optimization of the process operation and diagnosis of the fault in the bioprocess. Furthermore, up-scaling the bioreactor to the industrial scale is also essential, as it enables the translation of laboratory-scale production to commercial manufacturing.

#### 3.2.1. Monitoring and Control of Bioprocess

The operations in the biological process affect cell growth and product formation. Hence, monitoring the changes of important state variables in real-time, such as cell concentration and product concentration, is of great significance to optimizing the operations, the production culture and controlling the product quality. Due to the improvements in spectroscopic techniques and sensors, many advanced sensors have been applied for real-time monitoring of key process parameters in fermentation [117,118]. Most of these spectroscopic techniques require data processing and model setup, like Raman spectroscopy [118] and near-infrared (NIR) spectroscopy [119].

Raman spectroscopy with partial least squares regression (PLSR) is currently used for bioprocess monitoring, and has been applied in the mammalian cell (e.g., CHO cell lines) cultivations at both the lab scale and industrial scale [120,121]. Due to the time-varying, nonlinear and complex characteristics of the fermentation process, some key state parameters are difficult to measure in real-time by the existing sensors. Therefore, hybrid models combining kinetic models and machine learning methods are important tools to predict key parameters and construct soft-sensor models to further guide the optimization in industrial production processes. Zhang et al. constructed a hybrid model of artificial neural network and kinetics information with an automatic model structure identification framework [122]. They identified the optimal kinetic model structure to predict the key state variables, and optimize the production process of lutein from microalgae [122]. In the process of quality control of biotherapeutics, such as monoclonal antibodies, Antonakoudis et al. integrated a stoichiometric model with an artificial neural network to predict the glycosylation profile in CHO cell cultivations [123]. With this hybrid model, the glycan distribution profiles can be computed with accuracy and thus a platform is provided for process control in biotherapeutics production [123].

Many methods have been developed for soft-sensor modeling, and more details about the advanced methods can be found in the review of [124].

#### 3.2.2. Diagnosis and Analysis of Bioprocesses

Fault diagnosis is a technique that detects abnormal states occurring in production processes, which plays an important role in various biological fermentation processes. For example, Ding et al. constructed a fault diagnosis and rescue system based on a hybrid support vector machine and fuzzy reasoning to identify faults and their types at the earliest fermentation stage, and successfully applied them to glutamate fermentation [125]. By taking the relevant rescue measures based on the diagnosis results, the fermentations were successfully restored with the production of 75–80 g/L at 34 h [125]. Yang et al. proposed a hybrid model based on fast independent component analysis and probabilistic neural network (FICA-PNN) which could diagnose the faulty fermentation process in the fed batch production of penicillin more efficiently and accurately [126]. Abbsi et al. proposed a subspace-aided parity-based residual generation technique for fault detection and problem isolation in penicillin fermentation [127]. The method is based on the Just-In-Time (JIT) method which detects sensor faults and isolates and locates these problems [127]. This approach significantly improved the fault detection rate (FDR) and reduced the model complexity compared to existing diagnostic methods [127]. Yang et al. constructed a hybrid model for fault diagnosis and detection in penicillin fermentation by principal component analysis (PCA) for data dimensionality reduction, recursive feature elimination (RFE) for feature ranking and support vector machine (SVM) for the fault identification [128].

#### 3.2.3. Optimization and Scale-Up of Bioprocesses

Based on the real-time changes of key parameters in the process, we can optimize the cultivation media, feeding operation, etc., to maintain the cells in the optimal state and finally improve the production efficiency and product quality. Oyetunde et al. integrated genome-scale metabolic models (GEMs) with machine learning methods to assess the microbial bio-production by *E. coli* [129]. As an example, the key design features (such as reactor volume, temperature and media) of 1200 cell factories from over 100 literature studies were extracted and then ranked to determine the most important factors by PCA [129]. The features selected affected the microbial cell production performance with the constrained GEM iML1515 model [129]. This framework is capable of predicting metabolic changes under different conditions and effectively identifying the indicators for *E. coli* production performance [129]. Pinto et al. constructed a hybrid semi-parametric model by integrating kinetic models with machine learning methods to optimize the biomass growth setpoint, temperature and biomass concentration at induction in the fed-batch fermentation in *E. coli* [130]. They successfully optimized the cell growth and recombinant protein expression conditions [130]. Bayer proposed a bioprocess digital twin used for hybrid-model based DoE (design of experiment) to identify optimal process critical process parameters (CPP) by a minimum number of variables with the highest space-time yield in *E. coli* [131]. Additionally, to control the physical and chemical parameters (such as pressure, pH, DO, etc.) in the bioreactor, Kiran et al. proposed a neural network-based model predictive controller (NNMPC) to regulate the feed rate of the substrate to control the carbon dioxide evolution rate and oxygen consumption rate in the continuous fed-batch fermentation in *Saccharomyces cerevisiae* [132]. Kim et al. proposed a two-stage control framework for the fed-batch fermentation by a kinetic model with a differential dynamic programming (DDP) to determine the optimal substrate feeding strategy [103].

Bioreactor scale-up is a critical step in bioprocess development. CFD can be employed to simulate flow field changes in industrial-scale bioreactors, while metabolic models can be used to predict the performance of cell growth and production in bioprocesses. Furthermore, machine learning algorithms can be leveraged to reduce computational costs. The integration of these approaches is crucial for the development of multi-scale hybrid models that can capture the spatial—temporal dynamics of bioprocesses. By using such models, bioprocess scale-up can be realized at minimal cost, thereby advancing the bioprocess development process [58]. For example, Kuschel et al. combined a CFD model with a cell cycle model of *Pseudomonas putida* KT2440 to predict the factors on the change of flow field and glucose gradients in a 54,000 L stirred tank reactor [133]. They explored the effects of culture process conditions on the formation of population heterogeneity in large-scale production from the perspective of cell growth and energy requirements [133]. Bayer et al. established a hybrid model with the integration of ANNs and a kinetic model of CHO cells to predict the viable cell concentrations, and product titers at shake flask (300 mL) scale and 15 L bioreactor scale [134]. This model can identify critical process parameters (CPPs) rapidly and determine the transferability of DoE along process scales with an intensified Design of Experiments (iDoE) [134]. Liu et al. combined a CFD model and cell death dynamics to investigate the effect of shear effect of *C. tinctorius* L. cells in a 5 L bioreactor, and successfully improved the design and optimization of the cultivation in scale-up process [68]. Yeoh et al. investigated the spatial and temporal effects of mass and gas transfer in the reactor on cell growth and production by integrating a kinetic model of *E. coli* with a CFD model, effectively increasing the bioconversion to 94% from ferulic acid to vanillin [135].

## 4. Challenges and Future Perspectives

### 4.1. Challenges

Although the multi-omics analytics tools have become mature, we can obtain various layers of omics data. Nevertheless, it is still a challenge to extract information and knowledge from large omics data, and thus to optimize the bioprocess. Genomics, transcriptomics, proteomics, metabolomics and other omics data are high-dimensional with complex interrelationships. In-depth analysis of these data requires expertise and powerful computational capacity. However, most multi-omics analysis relies on manual screening to find the target of interest inefficiently. Additionally, the quality of these data is variable due to the lack of standardization in assessments. Different research teams have established various platforms to publish their results in different data formats, leading to challenges in data standardization and consistency. Furthermore, the pooled data analysis in the early stage of model construction is hindered by inconsistent data formats from multiple sources. Additionally, due to the lack of biological mechanistic data, a large deviation exists between production at a laboratory scale and at an industrial scale. Although many bioprocess sensors and soft-sensor methods have been developed to monitor the key parameters like pH, temperature and biomass, there would be no standard methods to monitor some state parameters such as biomass/product yield and productivity online. Due to the time intervals between offline data (some state parameters) and online data, the metabolic changes are hardly observed through these key state parameters, which will reduce the accuracy and completeness of model construction. The quality and reliability of data obtained in actual industrial production cannot be guaranteed, so the models based on historical production data are difficult to apply in practical production. In the upstream design of biological development process, researchers have developed rational models to meet different requirements from engineered strain design to optimization and scale-up of bioreaction. Nevertheless, methods for the construction of multi-scale hybrid models that associate the dynamic cellular growth with the continuous environmental changes have been less studied.

At present, many models have been established for the optimization of process operations in upstream bioprocess development, but most of them are applied to laboratory-scale production or even only remain in a theoretical stage. This is partly because the hybrid models based on CFD models require a high computational capacity to simulate the complex biological process and flow field characteristics in the bioreactors. The computational cost to simulate such a complex model exceeds the computational upper limit. Furthermore, the instruments capturing the nutrient, product and metabolite changes in the environment, such as Raman spectroscopy and infrared spectroscopy, cannot be directly applied in industrial production. In this case, the key factors that affect cellular metabolisms cannot be accurately determined at industrial scale.

### 4.2. Future Perspectives

To fully leverage biological process data, it is essential to standardize data acquisition methods. To maximize the potential of biological process data, it is crucial to develop unified protocols for data collection, ensuring consistency and comparability across different studies. By adopting standardized approaches, researchers can effectively integrate data from various sources, enabling comprehensive analyses and meaningful comparisons. Additionally, the utilization of cloud computing and big data technologies enables efficient storage, management, and retrieval of large-scale, multidimensional data, thereby enhancing data management and storage capabilities.

Exploring and developing data analysis methods as well as modeling techniques is vital to uncovering potential patterns and relationships within the data. Integration of machine learning, artificial intelligence and other methods can facilitate data integration and model construction, providing a deeper understanding of biological processes. Currently, research teams have already constructed advanced tools for multi-omics deep analysis [136,137,138]. Machine learning algorithms such as PLS, CNN and GNN are utilized to analyze the data mentioned above and integrate them into the models.

For the bioprocess, models have been established for the cell growth and production processes of various organisms, from prokaryotes like *Escherichia coli* and eukaryotes like *Saccharomyces cerevisiae* to mammalian cells, which play a vital role in bioprocess development in all aspects. As a bridge to intellectualize, modeling serves a significant role in linking digitalization to intellectualization. Multi-scale biological process data can effectively improve model accuracy. For instance, multi-constraint genome-scale metabolic models integrate multi-omics data and further construct the whole-cell models which demonstrate the changes in cellular metabolism to the greatest extent. The construction of such hybrid models requires detailed large biological metabolic data. Nowadays, it is possible to monitor the metabolic changes in the fermentation process in real-time at a laboratory scale with the help of fluorescence probe, real-time microscope and other advanced sensors.

In the process of model setup, cellular metabolism can be coupled with environmental changes to further analyze the phenotype changes across multiple bioreactor scales. Representatively, hybrid models coupling the CFD dynamics with cellular kinetics are amenable to identifying the main metabolic changes among different production scales. Such hybrid models can greatly reduce the manpower burden associated with experimental design and validation in the early stage of bioprocess development, contributing to the improvement of the automation level of process. Additionally, with the help of high-throughput screening devices, microfluidic technology, industrial robotic arms and automatic robots, the intelligent regulation of the production process can be eventually achieved, accelerating the establishment of a smart factory.

## Data Availability

Not applicable.
